# The Late Professor Harrison

**Published:** 1850-01

**Authors:** 


*The late Professor Harrison.*—John Pollard Harrison was born in the city of Louisville, June 5th, 1796, and was therefore, in the 54th year of his age. His medical studies commenced in the city of Louisville, but the principal part of his pupilage was spent in the office of Dr. Chapman, of Philadelphia. He graduated in the University of Pennsylvania, in the spring of 1819; and immediately on his return to Louisville commenced practice. Dr. Harrison continued the practice of his profession, in the city of Louisville, in a successful and highly creditable manner, until 1835, a period of sixteen years, when he removed to Philadelphia. During his residence in Louisville, Dr. Harrison for several years held the office of physician to the Louisville Marine Hospital, in which he occasionally delivered clinical lectures. Shortly after his removal he accepted the Professorship of Materia Medica in the Medical Department of Cincinnati College, and immediately removed to this city. He remained in the Cincinnati College until its suspension, in 1839, when he retired to the private duties of his profession. In 1841, he was elected to the chair of Materia Medica. in the

Medical College of Ohio; and in 1847 he was transfered to the chair of Theory and Practice in the same school. In 1849, after having delivered two courses on Theory and Practice, he was restored, at his own suggestion, to the chair of Materia Medica. It will thus be seen that he discharged the duties of a professor for a period of twelve years.

The deceased held several important and honorable positions in different medical associations; he was elected president of the Medical Convention of Ohio, which met at Lancaster, in 1843; he was appointed by the American Medical Association, which assembled in Baltimore, May, 1848, chairman of the committee on Medical Literature, and he was elected Vice President of that body, at its recent meeting in Boston.

Professor Harrison was a ready and copious writer, which is abundantly attested by his numerous contributions to medical journals. During his connection with the Cincinnati College, he was one of the editors of the *Western Journal of Medicine*; and since that period he has contributed many articles to periodicals, chiefly, however, to the *Western Lancet*. The *first* original communication published in the Lancet was from his profilic pen, and his contributions occupied a conspicuous place in every volume of that periodical. In 1847, Professor Harrison became associated in the editorial management of the Lancet, which connection was continued until the period of his decease.

In addition to journal articles, Professor Harrison was the author of numerous public lectures, mostly introductories to his courses of medical instruction. His public lectures were always spirited and eloquent, his subjects well chosen and amply discussed—qualities which seldom failed to please an audience, and which was evinced by the almost uniform publication of his discourses. We presume that his published lectures are more numerous than those of any other professor in the United States for an equal number of years.

The more permanent works written by Professor Harrison, consist of a small volome entitled, "Essays and Lectures on Medical Subjects," published in 1835; and his larger work on Materia Medica, consisting of two volumes, published in 1845. These works evince a profound acquaintance with the subjects upon which they treat, which would do honor to any author. One of the best articles, according to our judgment, ever written by Professor Harrison, is an essay entitled, "Medical Experience," published in the volume of essays above alluded to.

In all of the positions in which Professor Harrison was placed, whether as a writer or lecturer, he manifested an enthusiasm, energy and capacity surpassed by few in our profession. His mind was well stored with general information; and in the profession of medicine his reading was most extensive and profound. Endowed with quick perceptive faculties, a strong memory and untiring industry, he could not fail to acquire a vast fund of knowledge. We have never met with a physician who retained a more vivid recollection of the sentiments and language of authors, and whose ability was greater to quote, at any moment, and to almost any extent, the views of nearly every prominent writer in our profession.

Professor Harrison was, in every sense, a *physician*. He was not a mere *trader* in medicine; but, imbued with a lofty and expanded view of the profession, he rose altogether above every mercenary consideration, and was actuated alone by the purest motives and the most exalted sentiments.

He never sacrificed principle to expediency, nor duty to interest; but with an exalted purity and iron strength of purpose, his course was always guided by the dictates of truth, honor and justice. Dissimulation was to him unknown; fraud and deception he knew not how to practice; he never betrayed a friend, nor sought to injure a rival. In his immediate profession he was truthful to a most rigid and inflexible degree; no love of gain or hope of applause could ever tempt him to violate those great laws of honor and fidelity which should ever pervade the conduct of the true and faithful physician.

As a high toned and honorable gentleman, a consistent and exemplary Christian, Professor Harrison was equally distinguished. His sphere in social life, and in the Presbyterian church, of which he was an active member, was wide and influential. For thirty years he had been a professed Christian; and in his dying moments, when the dark shadows of eternal night were fast gathering around his pillow, his Christian hope shone brightly, and his aspiration ascended to the skies.—*Western Lancet.*

